# Saccade latency can indicate temporal attention differences in ADHD

**DOI:** 10.1192/j.eurpsy.2025.2184

**Published:** 2025-08-26

**Authors:** L. R. R. Carreiro, T. O. dos Santos, B. C. Guedes, L. C. V. Pereira

**Affiliations:** 1 Universidade Presbiteriana Mackenzie, São Paulo, Brazil

## Abstract

**Introduction:**

Temporal attention (TA) is the ability to allocate attention to specific periods in time. Temporal processing is often impaired in individuals with ADHD, but additional studies are needed to expand knowledge about these deficits compared to control groups.

**Objectives:**

To analyze the performance of adults in TA tasks based on ADHD symptoms using eye-tracking to measure saccade latency.

**Methods:**

Forty young adults (20 meeting ADHD criteria and 20 in the control group - CG) performed an attentional task in which a fixation point (FP) was presented at the center of a computer screen; 1000 ms later, an arrow indicating right or left appeared. The task consisted of two blocks, one with a higher frequency of targets at a 400 ms interval and the other at a 1000 ms interval, among shorter and longer intervals. Participants were instructed to focus on the FP, prepare to respond to the higher frequency interval, and only move their eyes after the peripheral target appeared. Saccade latency—defined as the time between target appearance and the initiation of eye movement—was recorded using a Tobii eye-tracker. A repeated measures ANOVA was conducted for the analysis. Ethical approval was obtained.

**Results:**

For longer intervals, saccade latency was significantly different between the ADHD and control groups (p = .009). While the CG showed reduced latency, the ADHD group exhibited increased latency, demonstrating a diminished ability to control attention over time.

**Conclusions:**

Young adults with ADHD exhibit a reduced capacity to sustain attention over longer periods of time. **Grant:** CNPq process number: 408084/2021-9

**Disclosure of Interest:**

None Declared

